# Sodium Chloride and Its Influence on the Aroma Profile of Yeasted Bread

**DOI:** 10.3390/foods6080066

**Published:** 2017-08-12

**Authors:** Markus C. E. Belz, Claudia Axel, Jonathan Beauchamp, Emanuele Zannini, Elke K. Arendt, Michael Czerny

**Affiliations:** 1 School of Food and Nutritional Sciences, University College Cork, National University of Ireland, College Road, T12 Y337 Cork, Ireland; belz.markus@web.de (M.C.E.B.); c.axel@umail.ucc.ie (C.A.); e.zannini@ucc.ie (E.Z.); 2 Department of Sensory Analytics, Fraunhofer Institute for Process Engineering and Packaging IVV, 85354 Freising, Germany; jonathan.beauchamp@ivv.fraunhofer.de (J.B.); michael.czerny@ivv.fraunhofer.de (M.C.)

**Keywords:** descriptive sensory, PTR-MS, GC-MS, Ehrlich pathway, bread aroma, salt reduction chemical compounds: 2-phenylethanol (PubChem CID: 6054), (E)-2-nonenal (PubChem CID: 5283335), 2,4-(E,E)-decadienal (PubChem CID: 5283349)

## Abstract

The impact of sodium chloride (NaCl) concentration on the yeast activity in bread dough and its influence on the aroma profile of the baked bread was investigated. Key aroma compounds in the bread samples were analysed by two-dimensional high-resolution gas chromatography-mass spectrometry in combination with solvent-assisted flavour evaporation distillation. High-sensitivity proton-transfer-reaction mass spectrometry was used to detect and quantify 2-phenylethanol in the headspace of the bread dough during fermentation. The analyses revealed significant (*p* < 0.05) changes in the aroma compounds 2-phenylethanol, (E)-2-nonenal, and 2,4-(E,E)-decadienal. Descriptive sensory analysis and discriminating triangle tests revealed that significant differences were only determinable in samples with different yeast levels but not samples with different NaCl concentrations. This indicates that a reduction in NaCl does not significantly influence the aroma profile of yeasted bread at levels above the odour thresholds of the relevant compounds, thus consumers in general cannot detect an altered odour profile of low‑salt bread crumb.

## 1. Introduction

Sodium chloride (NaCl), or salt, is a major taste contributor to food. A reduction of salt in food products generally leads to less intense taste and flavour. The impact of salt reduction on taste profiles has been demonstrated for numerous foods, amongst them white yeasted bread. An early investigation on white yeasted, rye and rye-sourdough breads indicated a low consumer preference for the reduced-salt breads [1]. In contrast, however, Wyatt [2] observed no significant difference in consumer preference for white bread containing 50% less NaCl than a reference bread. The current challenge for food producers is to develop products with a reduced salt content but an unimpaired and consistent taste. This has been investigated with the use of salt replacers such as potassium chloride, magnesium chloride, ammonium chloride, calcium chloride and calcium carbonate [3,4], the use of sourdough [5], the inclusion of flavour-enhancing acids and other potent aroma compounds [6,7], or by changes to the bread crumb texture that influence the saltiness perception [8,9,10]. In contrast to taste, less is known about the influence of salt reduction on the volatile aroma profile of food. Volatile aroma compounds impart flavour to food, and the volatile fraction of bread is highly complex with about 600 volatile compounds reported to be present in bread crumb [11]. In particular, the yeast metabolism plays a key role in the development of the aroma profile of bread, and salt, primarily its sodium ions, have a direct impact on yeast activity. In addition to ethanol and carbon dioxide, many low-molecular-weight flavour compounds such as further alcohols, aldehydes, acids, esters, sulphides and carbonyl compounds are produced by the yeast metabolism. These volatile compounds are essential contributors to the flavour of fermented foods and beverages [12,13]. The Ehrlich pathway is one of several routes responsible for the generation of aroma compounds by yeast in bread. In particular it leads to the formation of potent compounds such as fusel alcohols and acids. The efficacy of the Ehrlich pathway in converting amino acids into alcoholic odorants was investigated, amongst others, by Czerny and Schieberle [14], who used stable isotope dilution assays (SIDAs) to demonstrate the conversion of ^13^C (6)-leucine to the metabolite 3-methylbutanol.

The reducing activity of yeast during bread dough fermentation also has a critical impact on bread aroma, as has been similarly observed during beer wort fermentation [15]. Unsaturated aldehydes such as (E)-2-nonenal and 2,4-(E,E)-decadienal are derived from the oxidation of linoleic acid and are well-known for their contribution to fatty odours in wheat bread [11]. The reduction of these unsaturated volatile compounds by yeast to their corresponding alcohols has an impact on bread aroma; as such, a variation in yeast activity results in an altered aroma of the bread. Notably, it has been observed that *Saccharomyces cerevisiae* fully reduces unsaturated aldehydes to the corresponding alcohols [16].

The present work aimed at determining the impact of NaCl on the yeast metabolism in bread dough during fermentation and its influence on the overall aroma of the bread. More specifically, the unsaturated aldehydes (E)-2-nonenal (fatty) and 2,4-(E,E)-decadienal (fatty), and the alcoholic compound 2-phenylethanol (rose-like) were investigated as the key aroma compounds in bread based on preliminary analyses and supported by reports in the literature [11,17,18]. Recently Martins et al., 2015 evaluated the impact of bread fortification with dry spent yeast from brewing industry on physical, chemical and sensorial characteristics of home-made bread with the goal of increasing its β-glucan content. The sensory analysis showed how only the key odour hexanal was presented a significant increase in fortified bread [19]. A complementary analytical approach using two-dimensional high-resolution gas chromatography-mass spectrometry (2D-HRGC-MS) and proton-transfer-reaction mass spectrometry (PTR-MS) was used to quantify and monitor the generation of the selected aroma compounds in bread crumb samples containing different levels of NaCl and yeast. Sensory analysis was performed on the bread crumb samples to determine their odour characteristics and assess the impact of salt reduction on bread crumb based on a discrimination triangle test.

## 2. Materials and Methods

### 2.1. Microbiology

Instant active dry yeast consisting of living cells of *Saccharomyces cerevisiae* (Panté; Puratos, Belgium) was diluted in Ringer solution at a concentration of 10^−5^ g mL^−1^. An aliquot of 10 µL of the yeast solution was grown as a centre colony on yeast-selective potato dextrose agar plates (Fluka Chemie AG, Buchs, Switzerland) containing different amounts of sodium chloride (NaCl) (0, 0.3%, 1.2%, 2.0%, 3.0%, and 4.0% w/w) at 30 °C for 8 days. The growth rate was recorded every day by measuring the diameter of the visible colonies with an electronic calliper. 

### 2.2. Baking Procedure and Loaf Analyses

Wheat bread was prepared by mixing (spiral mixer, Kenwood KM020, Kenwood Manufacturing Co., Ltd., Hampshire, UK) Baker’s flour (Odlums, Dublin, Ireland), yeast, NaCl (Glacia British Salt Limited, Middlewich, UK) at levels of 0, 0.26%, 1.04%, 1.73%, 2.60% and 3.46% (w/w) and tap water (water levels set to 500 Brabender units, BU, depending on the amount of NaCl by using a farinograph, Brabender OHG, Duisburg, Germany). Considering an average bake loss of 13.5% the NaCl concentrations in the final bread loaves resulted in 0, 0.3%, 1.2%, 2.0%, 3.0% and 4.0% (Table 1). For each of the 10 different batches 6 loaves were prepared. For dough samples with varying amounts of yeast (1.5%, 0.9%, 0.6%, 0.3%), the concentrations of water and NaCl relative to the mass of flour were kept constant at 61.75% and 0.26% (w/w), respectively (Table 1). After bulk fermentation for 15 min at 30 °C and 85% relative humidity, bread loaves (450 ± 1 g) were formed using a molding machine (Machinefabriek Holtkamp B.V., Almelo, The Netherlands). The loaves were then placed into non-stick baking tins (180 mm × 120 mm × 60 mm), fermented for 75 min at 30 °C and 85% relative humidity, and then baked for 35 min at 230 °C (top and bottom heating). The ovens were pre-steamed (0.3 L water) and then steamed when loaded (0.7 L water). After baking, the loaves were removed from the tins and left to cool on cooling racks for 120 min at room temperature. Bake loss and specific volume were measured for all of the baked loaves. The bake loss was determined as the difference in mass between the dough and baked loaf. The specific volume was determined by a 3D laser scan using a VolScan Profiler 300 (Stable Micro Systems, Godalming, UK).

### 2.3. Rheofermentometer

A rheofermentometer (RheoF3, Chopin Technologies, Villeneuve-la-Garenne, Paris, France) was used to evaluate carbon dioxide (CO_2_) release and dough development of the different dough samples. Samples of 300 g of each dough were prepared in the same manner as described above for baking trials. The tests were performed at 30 °C over a 90 min period. As common practice for wheat dough, a cylindrical weight of 1.5 kg was applied onto the fermentation chamber. The total volume of CO_2_, the volume of retention for each sample, the lost volume of CO_2_, and the retention coefficient (capability of a dough to retain gas) were determined. Results are presented as the average of triplicate measurements.

### 2.4. Extraction of Volatile Aroma Compounds

Samples were prepared by cutting the bread crumb into 1 cm^3^ cubes, freezing these in liquid nitrogen, and then grinding them using a standard blender. Isotope-labelled standard solutions ([^2^H_2_]-(E)-2-nonenal 0.24 μg/mL; [^2^H_2_]-2,4-(E,E)-decadienal 0.50 μg/mL; [^2^H_4–5_]-2-phenylethanol 10.30 μg/mL) were added as internal standard to 50 ± 1 g of the ground crumb and the aroma compounds were extracted with 150 mL dichloromethane that was stirred at 120 rpm for 60 min at room temperature and then filtered to remove the suspension. This extraction step was repeated twice for each 50-g crumb sample and the filtrates were combined. The extracts were purified using solvent-assisted flavour evaporation (SAFE) distillation [20]. The distillates were concentrated down to a volume of 0.1 mL and these were subsequently stored at −20 °C prior to the analysis.

### 2.5. Two-Dimensional High-Resolution Gas Chromatography-Mass Spectrometry (2D-HRGC-MS)

Quantification of the selected aroma compounds was made using a two-dimensional high-resolution gas chromatography-mass spectrometer (2D-HRGC-MS), with a cryogenic trapping system (CryoTrap; Gerstel, Stadt, Germany) connecting the first GC system (Type 3800, Varian, Darmstadt, Germany) with a preparative DB-5 column to the second GC with a DB-FFAP column (each 30 m × 0.32 mm, 0.25 µm film thickness). The helium carrier gas flow was set to 1.5 mL min^−1^. The initial temperature of the first GC oven was 40 °C, which was subsequently heated at a rate of 8 °C min^−1^ to 230 °C. The eluting aroma compounds were transferred at defined retention times onto the cryo-trap, which was cooled to −100 °C. The volatiles were then flushed onto the column in the second GC oven by thermal desorption at 250 °C. The temperature of this second oven was increased from 40 °C to 250 °C at a rate of 6 °C min^−1^ and then held for 5 min at 250 °C. The eluting compounds were analysed with a Saturn 2200 mass spectrometer (Varian, Darmstadt, Germany) by chemical ionisation (CI) using methanol as the reagent gas.

### 2.6. Proton-Transfer-Reaction Mass Spectrometry (PTR-MS)

A high sensitivity proton-transfer-reaction mass spectrometer (hs-PTR-MS; IONICON Analytik GmbH, Innsbruck, Austria) was used to analyse the release of selected aroma compounds from the breads during fermentation. The instrument was operated at an electric field to buffer gas number density ratio (E/N) of 132 Td, which was established with drift tube settings of 600 V, 2.2 mbar and 60 °C. The PTR-MS was set to measure in mass scan mode in the range m/z 20–130 at a dwell time of 500 ms per m/z. Five individual scans of 51 s duration were made per sampling period, resulting in a complete analysis time of 255 s. A 1-m long, 1/16” OD, 0.04” ID Silcosteel™ (Restek GmbH, Bad Homburg, Germany) sample inlet line, heated to 65 °C and with a flow of 500 mL min^−1^, was used to transfer the sample gas to the instrument reaction chamber. 

Dough samples of 300 g were placed in 1-L perfluoroalkoxy (PFA) containers (AHF Analysentechnik GmbH, Darmstadt, Germany) for the on-line measurement of volatiles in the headspace of the dough during fermentation at 30 °C and 85% RH over 75 min. Five scan cycles of zero-air—i.e., air free of volatile organic compounds (VOCs)—in the empty sample container were made at the beginning of each analysis to determine the background noise and the limit of detection of the system. The mean signals from these scans were subtracted from the sample signals to correct for this background.

The intensities of the m/z relating to the abundance of the selected aroma compounds in the headspace gas of the sample chamber were converted to approximate concentrations (with an estimated accuracy of ± 30%) using a standard reaction rate (k) of 2.0 × 10^−9^ cm^3^/s [21]. The data were screened for m/z 105 specific to 2-phenylethanol, which is ionised to a cation by dehydroxylation and protonation. For the unsaturated aldehydes, the respective molecular ions at m/z 141 for (E)-2-nonenal and m/z 153 for 2,4-(E,E)-decadienal were outside the m/z scan range and thus could not be detected. It might be noted that a certain degree of fragmentation of these two compounds is expected to occur, thus their potential detection at m/z values within the scanned range might have been achievable, but this was hindered by a lack of knowledge of the exact fragments or their potential overlap with other compounds, and instrumental problems that impaired the sensitivity to m/z in the higher range. Thus only 2-phenylethanol will be reported for the PTR-MS data here.

### 2.7. Sensory Analysis

Sensory analyses of the samples were performed via the aroma profile analysis (APA) technique. Descriptive analyses were performed using a trained panel of 15 members, with at least ten assessors participating in each individual sensory session. The panellists were trained in weekly sessions to recognise the selected aroma compounds according to their odour qualities by smelling reference aqueous aroma solutions at different odorant concentrations. Training was performed over a period of at least six months prior to participation in the actual sensory experiments and the performance of each panellist was assessed via standard procedures. 

Bread loaves were cut into 2 cm-thick slices and the crust was removed. The yeasted dough and wheat flour bread samples were presented to the sensory panel for orthonasal assessment after storage in a closed glass beaker for 30 min at room temperature. The perceived odour qualities of the bread crumbs were described as being yeast/dough-like and flour-like based on a comparison to aqueous reference solutions. The panel agreed on the characteristic odour attributes of each sample in a group discussion. The pure compounds used for the reference solutions were purchased from Sigma-Aldrich (Taufkirchen, Germany), Acros (Geel, Belgium) and AromaLab (Freising, Germany). Crumb samples were then presented again to the panel in a second sensory session to evaluate the intensities of the aforementioned odour attributes on a scale from 0 (not detectable) over 1 (weak intensity), 2 (medium intensity) to 3 (high intensity). The sensory score of each attribute was calculated as an arithmetic mean. The assessors were trained immediately prior to the analysis with aqueous odorant solutions at defined super-threshold concentrations (factor 100 above the odour threshold) [18,22].

Sensory triangle tests were additionally performed on selected sample pairs by the panel to determine whether potential odour changes are detectable by consumers. The sample pairs, namely 0.3% and 1.5% yeast, 0.3% and 1.2% NaCl, and 1.2% and 3.0% NaCl were chosen based on a ‘standard-salt’ level of 1.2% w/w NaCl [23], a “low-salt” level of 0.3% w/w NaCl, and an “high-salt” level of 3.0% w/w NaCl. The bread loaves were sliced and uniform round pieces were punched-out for presentation. The panellists were required to smell the samples and identify which of the three samples differed (forced-choice test). The tests were repeated for all combinations of each sample pair during each of the three independent sessions. 

### 2.8. Statistical Analysis

Statistical analyses were performed on the sensory assessment results using Minitab for Windows statistical analysis software package (Systat Software, Inc., Chicago, IL, USA). The data were subjected to one-way analysis of variance (ANOVA). A Fisher’s least significant difference (LSD) test was performed for multiple comparisons for cases when an *F*-test showed significant differences (*p* < 0.05). Mean values of the three separate experiments with three independent samples from each batch were then calculated and are presented here, unless otherwise stated. The significance level was set to *p* < 0.05 throughout, unless otherwise stated.

## 3. Results and Discussion

### 3.1. Microbiology and Rheofermentometer

Initial assessments of the impact of NaCl on the activity and proliferation of the yeast showed an exponential inhibition of yeast proliferation with increasing amounts of NaCl. These results reflect previously reported effects of NaCl on yeast. Increased amounts of NaCl in the growth environment reduce the number of viable yeast cells as well as the biomass of the culture, while the length of the lag phase is increased [24,25,26,27]. The most important metabolite of yeast in bread dough is CO_2_, which leavens the dough and increases the volume of the bread loaf; thus, CO_2_ can also be used to monitor the yeast activity in dough. Table 2 lists the parameters relating to the yeast performance in wheat dough during fermentation; as can be seen, the total volume of CO_2_ produced decreased with increasing amounts of NaCl. The higher the amount of NaCl, the higher the osmotic pressure on the yeast cells, leading to growth inhibition and an inhibitory impact on the yeast metabolism [24,25]. The non‑significant difference between bread dough containing 0% and 0.26% NaCl is due to the NaCl threshold concentration required before conditions act to have an inhibitory influence on yeast [27,28]. The higher the NaCl level, the higher the retention coefficient, which leads to a higher retention of CO_2_ by the dough, as previously reported [23]. A lower CO_2_ production results in a lower pressure on the membranes of the gluten network. In addition, there are several reports that gluten networks are strengthened by NaCl and can thereby retain more CO_2_ [23,29,30].

### 3.2. Specific Bread Loaf Volume and Bake Loss

The specific volume and bake loss of the baked bread loaves were determined as a standard quality parameter (Table 3). The specific loaf volume decreased significantly for increasing amounts of NaCl at a yeast level of 1.2%. The bread containing no NaCl did not show a significantly larger volume than the bread with 0.3% NaCl, despite its water level being the highest at 62.95% (Table 1).

Although the dough was mixed to a standard consistency of 500 BU, this observation might be explained by a weaker gluten network in the absence of NaCl [23,29,30]. The data from the rheofermentometer corroborate this hypothesis; the generation of a non-significant higher amount of CO_2_ did not lead to a greater bread volume. The higher the NaCl concentration, the smaller was the bread volume and surface area, and less water could evaporate, which again is supported by the rheofermentometer data. The bake loss expressed as a percentage of the water level for each of the individual bread loaves showed that small adjustments of the water level to achieve a dough consistency of 500 BU did not significantly influence the bake loss (Table 3). The breads with yeast levels ranging from 0.3% to 1.5% at a constant NaCl level of 0.3% w/w had the highest specific volume at 0.6%. At lower yeast concentrations, the amount of yeast did not produce sufficient CO_2_ to stretch the gluten network to its maximum. At yeast concentrations, higher than 0.6% the specific volume decreased with increasing amounts of yeast. Similarly, increasing amounts of yeast led to excessive fermentation and a resulting expansion of the gluten network, thereby increasing the loss of CO_2_ [23,25,27]. 

### 3.3. Analyses of Volatile Aroma Compounds in Bread Crumb

Three key aroma compounds in wheat bread crumb are the alcoholic compound 2-phenylethanol [14,31] and the unsaturated aldehydes (E)-2-nonenal and 2,4-(E,E)-decadienal [16,32]. These three compounds were analysed in the aroma extracts of bread crumb by 2D-HRGC-MS (Table 4). The 2-phenylethanol decreased in concentration exponentially with increasing amounts of NaCl, but increased significantly with increasing amounts of yeast, albeit not for the highest yeast concentrations (0.9%, 1.2% and 1.5% w/w).

The concentrations of (E)-2-nonenal differed significantly for 3% and 4% NaCl compared to the lowest NaCl concentrations of 0%–0.6%. Increasing the yeast content led to a significant decrease in (E)-2-nonenal for the samples containing 0.3% and 0.6% yeast compared to the samples containing 0.9% and 1.5% yeast. Here, 2,4-(E,E)-decadienal did not show any significant change for the different NaCl concentrations, but decreased with increasing yeast content. These changes can be explained by the reducing activity of the baker’s yeast. Decreasing the yeast concentration or inhibiting the yeast with more salt lowers the overall yeast activity during the fermentation process, thereby resulting in a lower production of the unsaturated aldehydes. The reducing activity of yeast has been previously investigated [33]. Furthermore, Saison et al., 2010 demonstrated a change in beer aroma production based on the reducing activity of (E)-2-nonenal by *S. cerevisiae*, suggesting that the reducing activity metabolism of yeast is not affected by increased concentrations of NaCl in the same manner as the Ehrlich pathway or other parts of the yeast metabolism. Correlations (R^2^ ≥ 0.75) were found between CO_2_ and 2-phenylethanol or (E)-2-nonenal, indicating that these compounds must arise during yeast metabolism and are influenced by the amount of NaCl in bread dough. A negative correlation for the unsaturated aldehyde (E)-2-nonenal indicates that the reduction capacity of yeast increased with increasing yeast activity, thus an increased amount of (E)-2-nonenal was reduced to the corresponding alcohol.

### 3.4. PTR-MS Analyses of Bread Dough during Fermentation

**Figure 1:**
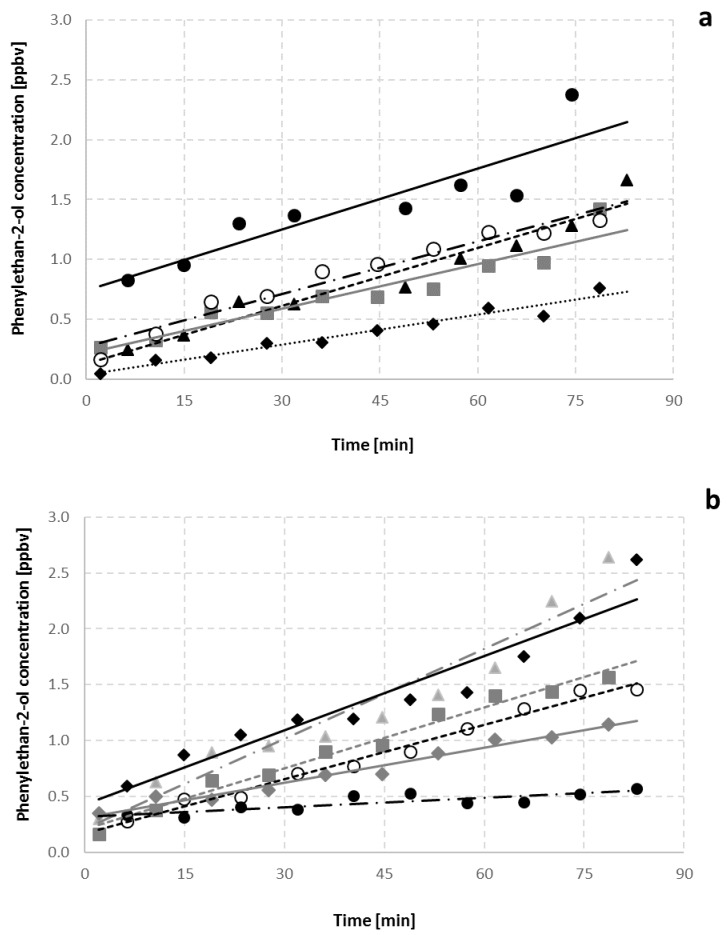
Volume mixing ratios of 2-phenylethanol in the headspace of bread dough containing (**a**) different yeast concentrations (w/w) of 1.5% (filled circles with solid regression line), 1.2% (open circles with black dot-dashed regression line), 0.9% (filled squares with grey regression line), 0.6% (filled triangles with dashed regression line), 0.3% (filled diamonds with dotted regression line) and (**b**) different NaCl concentrations (w/w) of 0.0% (filled triangles with grey dot-dashed regression line), 0.3% (filled diamonds with solid regression line), 1.2% (filled squares with grey dashed regression line), 2.0% (open circles with black dashed regression line), 3.0% (filled diamonds with grey regression line) and 4% (filled circles with dot-dashed regression line) over 90 min of incubation under fermentation conditions (30 °C, 85% RH).

The PTR-MS data for the rose-like aroma compound 2-phenylethanol at m/z 105 in the headspace of the dough showed that its concentration increased for increasing amounts of yeast (0.3%–1.5%), albeit with the regressions between 0.6 and 1.2% being similar (Figure 1a). The more NaCl that was added to the bread samples, the less 2-phenylethanol was present due to the inhibiting effect of NaCl on yeast (Figure 1b). The headspace concentrations of 2-phenylethanol correlate with the concentration of this compound in the respective bread crumb samples, as measured by 2D-HRGC-MS. 

### 3.5. Descriptive Sensory Evaluation

**Figure 2:**
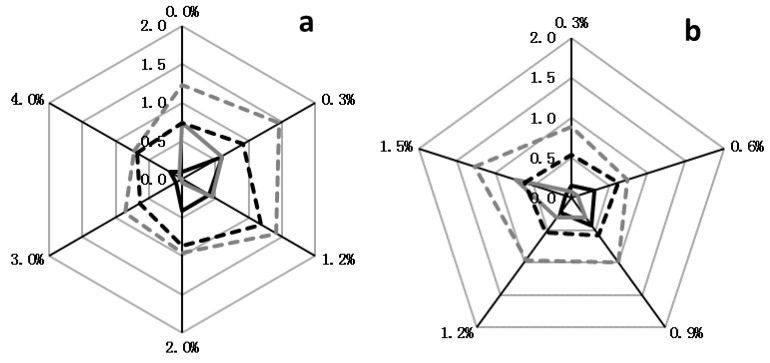
Aroma profiles of bread crumbs for the attributes *cheesy* (grey line), *rose-like* (black line), *fatty* (black dashed line) and *buttery* (grey dashed line) at (**a**) different NaCl concentrations (0.0%, 0.3%, 1.2%, 2.0%, 3.0% and 4% w/w) and (**b**) different yeast levels (0.3%, 0.6%, 0.9%, 1.2% and 1.5% w/w) on a scale from 0 (not detectable) over 1 (weak intensity) to 2 (medium intensity), as determined by sensory panel.

Ten sensory attributes were collected for the bread-crumb samples, whereby the dominating attributes were roasty, yeasty, malty, flour like, buttery, cheesy and fatty. The APAs of the bread-crumb samples revealed significant differences only for the attributes *cheesy* and rose-like (Figure 2). 

The *cheesy* aroma was significantly different between the samples with the three highest NaCl levels (2%, 3%, and 4% w/w) and the three lowest levels (0.0%, 0.3%, and 1.2% w/w NaCl) (Figure 2a). In the samples with varying yeast levels only the highest (1.5% w/w yeast) and lowest (0.3% w/w yeast) levels differed significantly with respect to the *cheesy* odour impression (Figure 2b). Butanoic acid is well-known as a volatile metabolic compound of yeast with a *cheesy* odour note, and is listed in the Yeast Metabolic Database (YMDB) ID 01392 [34]. The results of these APAs correlate with the yeast activity during fermentation. Increased amounts of yeast as well as decreasing NaCl levels result in higher yeast activities and hence, a higher metabolism rate, including production of the metabolite butanoic acid, which results in a more intense cheesy odour.

The attribute rose-like determined by the sensory panel (Figure 2) correlated directly with the concentration of 2-phenylethanol in the bread crumb samples (Table 4) and hence, with the yeast activity. Increasing yeast activity during dough fermentation led to higher concentrations of 2-phenylethanol. The lower the NaCl level or the higher the yeast level, the more intense was the rose-like odour, as determined by the sensory panel (Figure 2). At 0.0% w/w NaCl the panel did not perceive a rose-like odour, which might reflect a totally excessive and uncontrolled yeast activity in the complete absence of NaCl. Odour impressions described as cheesy and buttery dominated the overall odour characteristic and covered the rose-like impression (Figure 2). The same effect is shown for an increased amount of yeast above 0.9% w/w. The rose-like compounds, namely 2-phenylethanol and 2-phenylacetic acid, have higher odour thresholds compared to the other aroma compounds considered here, therefore the rose-like odour fraction can easily be dominated by other volatile aroma compounds or influence their recognition [22].The fatty impression in the samples with differing NaCl content did not vary significantly, as confirmed by 2D-HRGC-MS analyses of the unsaturated aldehydes (E)-2-nonenal and 2,4-(E,E)-decadienal, both of which have characteristic fatty odour impressions. By contrast, significantly different concentrations of both aldehydes were found in the samples of varying yeast content (Table 4), although the sensory panel could not differentiate between these samples. This might be due to an increasing yeast activity, which predominantly produces other volatile aroma compounds such as butanoic acid and Ehrlich pathway metabolites, which results in an increase in the buttery odour, as observed here (Figure 2).

### 3.6. Sensory Triangle Test

Sensory triangle test analyses of the two sample pairs with different NaCl levels indicated that there was no significant difference between the pairs (*p* > 0.2). The sample pair with 0.3% and 1.5% w/w yeast was significantly distinguishable (*p* < 0.1). These findings show that NaCl reduction has no significant influence on the overall volatile aroma fraction of the bread crumb recognisable by a human panellist. On the contrary, a 5-fold increase of yeast led to a distinguishable difference based on a 5-fold increase of the yeast metabolism activity in the dough system. The descriptive sensory results show several changes for isolated attributes and compounds in particular based on the determined attributes rose-like and cheesy. However, the aroma profile as a whole did not change to an extent that would be recognisable by consumers. 

## 4. Conclusions

The influence of yeast activity on the generation of volatile aroma compounds in bread crumb was investigated using sensory assessments in combination with two-dimensional high-resolution gas chromatography-mass spectrometry (2D-HRGC-MS) and proton-transfer-reaction mass spectrometry (PTR-MS). A correlation between different yeast metabolites was shown. The metabolic pathways in yeast cells seem to correlate with the reducing activity, independently of the amount of yeast present or the concentration of NaCl. Bread samples with a 5-fold increase in yeast concentration (from 0.3% to 1.5% w/w) were distinguishable by a trained sensory panel. Indeed, when the concentration of NaCl was kept at 0.3%, yeast cells were metabolically over-performing due to the lack of the inhibiting effect normally carried out by the presence of NaCl in the dough system. This related to an increased yeast metabolic activity during the fermentation process, which showed to have a significant impact on the final bread crumb aroma [17]. A reduction in NaCl from the standard concentration of 1.2% w/w to 0.3% w/w increased the yeast activity but the increase in volatile aroma components, as determined by 2D-HRGC-MS and PTR-MS, could not be detected by the sensory panel. These observations suggest that NaCl reduction does not influence the volatile aroma of bread significantly and consumers in general are not able to recognise any reduced amounts of salt in the odour of bread crumb. While salt reduction in bread impacts on the quality characteristics of taste, shelf-life and texture [22,35], the aroma quality remains unchanged.

## Figures and Tables

**Table 1:** Ingredient quantities in the breads containing varying amounts of NaCl at constant yeast (1.2% w/w) and varying amounts of yeast at constant NaCl (0.3% w/w) *.

Ingredient	Ingredient Quantity for Each Type of Bread (% w/w flour)									
	Variable NaCl (% w/w) at 1.2% w/w Yeast						Variable Yeast (% w/w) at 0.3% w/w NaCl			
	0	0.26	1.04	1.73	2.60	3.46	1.5	0.9	0.6	0.3
Wheat flour	100.00	100.00	100.00	100.00	100.00	100.00	100.00	100.00	100.00	100.00
Yeast	2.00	2.00	2.00	2.00	2.00	2.00	2.47	1.48	0.98	0.48
Tap water (30°)	62.95	61.75	61.00	60.90	59.75	59.75	61.75	61.75	61.75	61.75
NaCl	0.00	0.43	1.72	2.87	4.36	5.80	0.43	0.43	0.43	0.43

* The 0.3% NaCl concentration was used in accordance to the Directive 80/777/EEC to claim a bread low in salt.

**Table 2:** Yeast performance in wheat dough with different NaCl concentrations during 3 h fermentation.

NaCl Concentration (% w/w)	Total Volume of Dough (mL)	Volume of Retention (mL)	Volume of CO_2_ Lost (mL)	Retention Coefficient
0.00	2241 ± 54 ^a^	1435 ± 23 ^a^	806 ± 75 ^a^	64.1 ± 2.4 ^a^
0.26	2108 ± 40 ^a^	1459 ± 11 ^a^	649 ± 49 ^b^	69.2 ± 1.8 ^d^
1.04	1581 ± 126 ^b^	1337 ± 54 ^b^	243 ± 78 ^c^	84.8 ± 3.7 ^c^
1.73	982 ± 28 ^c^	953 ± 22 ^c^	30 ± 8 ^d^	97.0 ± 0.7 ^b^
2.60	573 ± 15 ^d^	569 ± 14 ^d^	4.0 ± 1.7 ^e^	99.4 ± 0.2 ^a^
3.46	313 ± 11 ^e^	311 ± 11 ^e^	2.0 ± 0.0 ^f^	99.4 ± 0.1 ^a^

Values in one column followed by the same superscript letter are not significantly different (*p* < 0.05).

**Table 3:** Specific volume and bake loss of bread loaves with different NaCl concentrations and standard yeast level of 1.2% w/w and with different yeast concentrations and standard NaCl concentration of 0.3% w/w.

Bread Type		Specific Bread Volume (mL/g)	Bake Loss *	
			(% w/w)	(% of Water Level)
Varying NaCl (% w/w) at 1.2% w/w yeast	0.0	3.85 ± 0.13 ^a^	14.9 ± 0.5 ^a,b^	39.1 ± 1.2 ^a,b,c^
	0.3	3.81 ± 0.08 ^a^	15.3 ± 0.3 ^a^	40.6 ± 0.8 ^a^
	1.2	3.49 ± 0.09 ^b^	14.4 ± 0.4 ^b^	38.6 ± 0.9 ^b^
	2.0	3.11 ± 0.05 ^c^	13.4 ± 0.5 ^c^	37.4 ± 0.6 ^c^
	3.0	2.52 ± 0.02 ^d^	12.3 ± 0.4 ^d^	32.7 ± 1.0 ^d^
	4.0	1.93 ± 0.03 ^e^	10.6 ± 0.3 ^e^	28.6 ± 0.8 ^e^
Varying yeast (% w/w) at 0.3% w/w NaCl	0.3	3.02 ± 0.02 ^i^	13.4 ± 0.2 ^g^	36.2 ± 0.5 ^g^
	0.6	3.70 ± 0.05 ^f^	14.7 ± 0.2 ^f^	39.8 ± 0.7 ^f^
	0.9	3.62 ± 0.19 ^f,g^	14.7 ± 0.2 ^f^	39.7 ± 0.6 ^f^
	1.2	3.49 ± 0.09 ^g,h^	14.4 ± 0.4 ^f^	38.6 ± 0.9 ^f^
	1.5	3.37 ± 0.10 ^h^	13.9 ± 0.5 ^f,g^	38.2 ± 0.9 ^f^

* The bake loss is shown as a percentage of the dough mass as well as a percentage of the water level. Values in one column followed by the same superscript letter are not significantly different (*p* < 0.05).

**Table 4:** Concentration of 2-phenylethanol, (E)-2-nonenal and 2,4-(E,E)-decadienal in bread-crumb samples as determined by 2D-HRGC-MS.

Bread Type		Concentration (µg/kg)		
		2-Phenylethanol	(E)-2-Nonenal	2,4-(E,E)-Decadienal
Varying NaCl (% w/w) at 1.2% w/w yeast	0.0	4441 ± 686 ^a^	12.9 ± 1.7 ^c,d^	12.7 ± 1.1 ^a^
	0.3	3055 ± 549 ^a^	13.5 ± 1.2 ^c,d^	11.1 ± 0.7 ^a^
	1.2	1582 ± 6 ^b^	12.1 ± 0.3 ^d^	8.5 ± 0.8 ^b^
	2.0	1685 ± 88 ^c^	15.9 ± 2.6 ^b,c^	9.2 ± 1.3 ^a,b^
	3.0	1189 ± 9 ^d^	16.4 ± 1.2 ^b^	10.6 ± 2.0 ^a,b^
	4.0	845 ± 58 ^e^	20.8 ± 2.7 ^a^	9.3 ± 0.7 ^a,b^
Varying yeast (% w/w) at 0.3% w/w NaCl	0.3	1164 ± 64 ^i^	19.7 ± 0.0 ^e^	20.2 ± 1.8 ^c^
	0.6	1773 ± 40 ^h^	16.5 ± 1.9 ^f^	17.9 ± 3.1 ^c^
	0.9	2607 ± 165 ^g^	10.2 ± 2.2 ^g,h^	11.4 ± 0.7 ^d^
	1.2	3056 ± 549 ^f,g^	13.5 ± 1.2 ^f,g^	11.1 ± 0.7 ^d^
	1.5	3288 ± 315 ^f^	8.0 ± 2.3 ^h^	8.6 ± 0.1 ^e^

Values in one column followed by the same superscript letter are not significantly different (*p* < 0.05).
